# Engineering *Saccharomyces cerevisiae* for the Overproduction of β-Ionone and Its Precursor β-Carotene

**DOI:** 10.3389/fbioe.2020.578793

**Published:** 2020-09-30

**Authors:** Javiera López, Diego Bustos, Conrado Camilo, Natalia Arenas, Pedro A. Saa, Eduardo Agosin

**Affiliations:** ^1^Centro de Aromas y Sabores, DICTUC S.A., Santiago, Chile; ^2^Department of Chemical and Bioprocess Engineering, School of Engineering, Pontificia Universidad Católica de Chile, Santiago, Chile

**Keywords:** *Saccharomyces cerevisiae*, ionone, apocarotenoid biosynthesis, metabolic engineering, synthetic biology

## Abstract

β-ionone is a commercially attractive industrial fragrance produced naturally from the cleavage of the pigment β-carotene in plants. While the production of this ionone is typically performed using chemical synthesis, environmentally friendly and consumer-oriented biotechnological production is gaining increasing attention. A convenient cell factory to address this demand is the yeast *Saccharomyces cerevisiae*. However, current β-ionone titers and yields are insufficient for commercial bioproduction. In this work, we optimized *S. cerevisiae* for the accumulation of high amounts of β-carotene and its subsequent conversion to β-ionone. For this task, we integrated systematically the heterologous carotenogenic genes (CrtE, CrtYB and CrtI) from *Xanthophyllomyces dendrorhous* using markerless genome editing CRISPR/Cas9 technology; and evaluated the transcriptional unit architecture (bidirectional or tandem), integration site, and impact of gene dosage, first on β-carotene accumulation, and later, on β-ionone production. A single-copy insertion of the carotenogenic genes in high expression *loci* of the wild-type yeast CEN.Pk2 strain yielded 4 mg/gDCW of total carotenoids, regardless of the transcriptional unit architecture employed. Subsequent fine-tuning of the carotenogenic gene expression enabled reaching 16 mg/gDCW of total carotenoids, which was further increased to 32 mg/gDCW by alleviating the known pathway bottleneck catalyzed by the hydroxymethylglutaryl-CoA reductase (HMGR1). The latter yield represents the highest total carotenoid concentration reported to date in *S. cerevisiae* for a constitutive expression system. For β-ionone synthesis, single and multiple copies of the carotene cleavage dioxygenase 1 (CCD1) gene from *Petunia hybrida* (*Ph*CCD1) fused with a membrane destination peptide were expressed in the highest β-carotene-producing strains, reaching up to 33 mg/L of β-ionone in the culture medium after 72-h cultivation in shake flasks. Finally, interrogation of a contextualized genome-scale metabolic model of the producer strains pointed to *Ph*CCD1 unspecific cleavage activity as a potentially limiting factor reducing β-ionone production. Overall, the results of this work constitute a step toward the industrial production of this ionone and, more broadly, they demonstrate that biotechnological production of apocarotenoids is technically feasible.

## Introduction

Isoprenoids are the largest and most diverse group of natural compounds found in nature. Many members of this family have attractive commercial applications in both the flavor and fragrance industries, finding place in cosmetics, perfumes and food formulations. An interesting subfamily of these compounds are plants apocarotenoids, highly valued by the food industry. These multifaceted compounds – produced by the enzymatic cleavage of carotenoids –encompass pigments, aromas and scent compounds, amongst others, with yet unknown functions.

One of the most interesting apocarotenoids are those of the family of ionones. Particularly, β-ionone – a highly valued ionone for its woody-violet aroma – is produced by the cleavage of the C40-compound β-carotene by the *carotenoid cleavage dioxygenase 1* (CCD1). This enzyme can symmetrically cleave the 9,10 (9’,10’) double bonds of multiple carotenoid substrates to produce a C14 dialdehyde and two C13 ionones (Vogel et al., 2008). Both molecules, β-carotene and β-ionone, have been heterologously produced by different microorganisms. In the case of β-carotene, a number of studies have reported heterologous β-carotene production by recombinant *Saccharomyces cerevisiae* strains in shake flasks (Yamano et al., 1994; Verwaal et al., 2007; Li et al., 2013; Zhao et al., 2015; Li et al., 2017), and bench-scale batch fermentations (Reyes et al., 2014; Olson et al., 2016; López et al., 2019), reaching up to 18 mg/gDCW in test tubes and 25 mg/gDCW in batch fermenters (Olson et al., 2016). All the above studies employed constitutive expression systems. On the other hand, Xie et al. (2015a) achieved 20.8 mg/gDCW in high-cell density fermentations after engineering an inducible expression system in recombinant yeast cells with a sequential control strategy (Xie et al., 2015b).

In the case of β-ionone, one of the first platforms used for its production was *S. cerevisiae*, reaching titers in the range of 0.2 to 5 mg/L (Beekwilder et al., 2014; López et al., 2015). More recent attempts by Werner et al. (2019) in two-phase fermentations in shake flasks, reported 180 mg/L of β-ionone – in the organic phase – using a β-carotene hyperproducer strain (Reyes et al., 2014), the highest titer reached to date in this yeast. Nevertheless, this strain has a poor performance under industrial fermentation conditions (López et al., 2019). Production in other microorganisms has also been attempted. For example, in *Escherichia coli*, titers of 32 mg/L in shake flasks and 500 mg/L in bioreactors have been reported (Zhang et al., 2018); while in the oleaginous yeast *Yarrowia lipolytica* its accumulation reached up to 60 mg/L and 380 mg/L of β-ionone in the same scales, respectively (Czajka et al., 2018).

Due to the low concentrations of carotenoids in plants [in the order of mg/100 g of fresh weight, Vogel et al. (2010)], it is expected that CCDs enzymes were not evolved to process high concentrations of their substrates (Chen et al., 2019). This is probably one of the reasons why in heterologous hosts, like *E. coli* and *S. cerevisiae*, CCD activity is suggested as the limiting production step (Czajka et al., 2018; Werner et al., 2019). The structure of CCD enzymes shows a conserved, seven-bladed β-propeller with a central tunnel, where a Fe^2+^ is located (Harrison and Bugg, 2014; Werner et al., 2019). A second conserved structural feature of these enzymes is located above the β-propeller, corresponding to a dome formed by loops and α-helices. This dome is a hydrophobic patch covering the enzyme’s surface, which interacts with the hydrophobic side of biological membranes (Sui et al., 2013). Efforts in the optimization of the catalytic efficiency of these enzymes by protein engineering have been mildly successful due to the high conservation degree of their structural features (Floss and Walter, 2009). For example, Ye et al. (2018) improved β-ionone production in *E. coli* by optimizing the localization of the catalytic enzymes, e.g., CCD1, according to the availability of the corresponding substrate, e.g., β-carotene. More recently, *S. cerevisiae* strains expressing constructs of *Ph*CCD1 fused with membrane destination peptides showed β-ionone yields up to 4-fold higher than the strain carrying the native enzyme (Werner et al., 2019).

The industrial bioproduction of β-ionone is still commercially infeasible due to the currently poor yields, productivities and titers. Metabolic engineering of the microbial host is critical to ensure reaching economically viable production targets. A widely used approach to achieve high titers of secondary metabolites in biofactories involves the expression of heterologous genes using high-copy number plasmids. This strategy is, however, inefficient in most cases, as it typically requires the use of selective media, causes genomic instability, and can impose a high metabolic burden to the cell (Ryan et al., 2014). Conversely, genomic integration of expression cassettes is a more convenient strategy, since the resulting recombinant strains are generally more stable, and their gene expression more controllable (Jensen et al., 2014; Amen and Kaganovich, 2017). Nevertheless, the limited number of selection markers, integration sites and genetic arrangements, constrain the strain design as they restrict the allowable gene dosage (copy number) for the construction. The development of CRISPR/Cas9 technology in yeast has minimized the need of selection markers and, most importantly, it has proven to be highly effective for *S. cerevisiae* transformation, owed to its high homologous recombination capability in response to double strand breakage (DSB) (Jessop-Fabre et al., 2016). Moreover, this technology enables simultaneous integration of several expression cassettes, thereby accelerating strain construction and optimization (Ryan et al., 2014).

**FIGURE 1:**
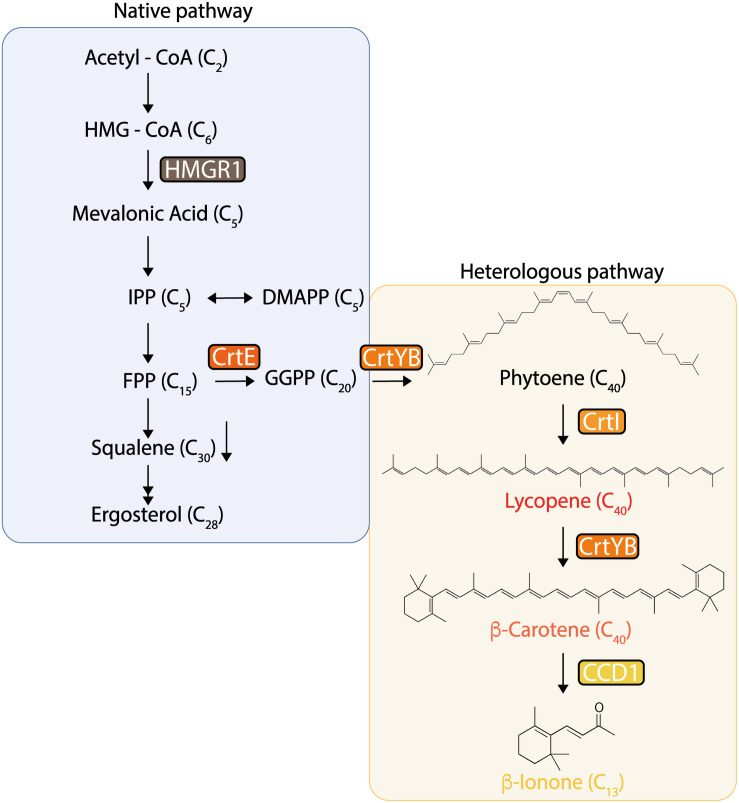
Native and heterologous pathways for β-carotene and β-ionone production in *Saccharomyces cerevisiae*. In these pathways, the *tHMG1* gene (encoding a truncated HMG-CoA reductase), and the heterologous genes *crtE* (encoding geranylgeranyl diphosphate synthase), *crtYB* (encoding phytoene synthase/lycopene cyclase), *crtI* (encoding phytoene desaturase), and *PhCCD1* (encoding carotenoid cleavage dioxygenase from *P. hybrida*) were constitutively integrated into the yeast’s genome in different locations, configurations, and dosage.

In this work, we constructed several *S. cerevisiae* strains using the CRISPR/Cas9 technology, capable of accumulating increasing concentrations of β-ionone, and its precursor, β-carotene. To this task, we first integrated the heterologous genes leading to β-carotene production into specific high-expression sites flanked by genetic elements that are essential for growth of the wild-type *S. cerevisiae* strain CEN.PK2-1c (Mikkelsen et al., 2012). These genes coded for the following enzymes: geranylgeranyl diphosphate synthase (CrtE), bifunctional phytoene synthase/lycopene cyclase (CrtYB), and phytoene desaturase (CrtI) (Figure 1). Integration of heterologous genes in these sites enabled robust expression of multi-gene constructs in few known *loci*, thereby avoiding genetic instability issues. The effect of both, the genetic constructs architecture and gene dosage, was then evaluated for generating high β-carotene producers. The most promising producers were later selected and transformed with an engineered CCD1 gene from *Petunia hybrida* (fyn-*Ph*CCD1) for β-ionone overproduction (Werner et al., 2019). Incremental increase of the gene dosage of fyn-*Ph*CCD1 up to a certain level enabled reaching the highest β-ionone titer reported to date in *S. cerevisiae* shake-flask cultures. Finally, a contextualized genome-scale stoichiometric model of the producer strains unveiled the potential high impact of *Ph*CCD1 inefficient activity for high β-ionone production.

## Results and Discussion

### Design and Evaluation of Different Transcriptional Unit Architectures and Integration Sites for Carotenoids Production

To efficiently produce β-carotene in *S. cerevisiae*, we first evaluated the impact of the arrangement of the carotenogenic (heterologous) genes and the chromosomal integration *loci*, on the final production yield. For this purpose, we constructed several carotenogenic strains expressing the genes CrtE, CrtYB and CrtI from the yeast *X. dendrorhous* (Table 1A), under three constitutive strong yeast promoters (TEF1, PGK1 and TDH3).

**TABLE 1:** List of strains constructed in this study.

(A) β-Carotene production			

Strain	Parental strain	Genotype	Integration site (Chr-Site)
CEN.PK2-1c	-	Mata ura3-52 trp1-289 leu2-3, 112 his3D	-
β-Car1.1/β-Car1.2	CEN.PK2-1c	P*_*TEF1*_*-***CrtE***-T*_*ADH1*_*/P*_*PGK1*_*-***CrtYB***-T*_*CYC*_*	XI-5
		P*_*TDH3*_*-***CrtI***-T*_*CYC*_*	XI-3
β-Car2.1	CEN.PK2-1c	P*_*TEF1*_*-***CrtE***-T*_*ADH1*_*/P*_*PGK1*_*-***CrtYB***-T*_*CYC*_*/P*_*TDH3*_*-***CrtI***-T*_*CYC*_*	XI-5
β-Car2.2	CEN.PK2-1c	P*_*TEF1*_*-***CrtE***-T*_*ADH1*_*/P*_*PGK1*_*-***CrtYB***-T*_*CYC*_*/P*_*TDH3*_*-***CrtI***-T*_*CYC*_*	XI-3
β-Car2.3	CEN.PK2-1c	P*_*TEF1*_*-***CrtE***-T*_*ADH1*_*/P*_*PGK1*_*-***CrtYB***-T*_*CYC*_*/P*_*TDH3*_*-***CrtI***-T*_*CYC*_*	X-2
β-Car2.4	CEN.PK2-1c	P*_*TEF1*_*-***CrtE***-T*_*ADH1*_*/P*_*PGK1*_*-***CrtYB***-T*_*CYC*_*/P*_*TDH3*_*-***CrtI***-T*_*CYC*_*	X-4
β-Car2.5	CEN.PK2-1c	P*_*TEF1*_*-***CrtE***-T*_*ADH1*_*/P*_*PGK1*_*-***CrtYB***-T*_*CYC*_*/P*_*TDH3*_*-***CrtI***-T*_*CYC*_*	XI-1
β-Car2.6	CEN.PK2-1c	P*_*TEF1*_*-***CrtE***-T*_*ADH1*_*/P*_*PGK1*_*-***CrtYB***-T*_*CYC*_*/P*_*TDH3*_*-***CrtI***-T*_*CYC*_*	XI-2
β-Car3	β-Car2.1	P*_*TEF1*_*-***CrtE***-T*_*ADH1*_*/P*_*PGK1*_*-***CrtYB***-T*_*CYC*_*/P*_*TDH3*_*-***CrtI***-T*_*CYC*_*	XI-3
β-Car4.a	β-Car3	P*_*TEF1*_*-***CrtE***-T*_*ADH1*_*/P*_*PGK1*_*-***CrtYB***-T*_*CYC*_*/P*_*TDH3*_*-***CrtI***-T*_*CYC*_*	X-2
β-Car4.b	β-Car3	P*_*TEF1*_*-***CrtE***-T*_*ADH1*_*/P*_*PGK1*_*-***CrtYB***-T*_*CYC*_*	X-2
β-Car5	β-Car4.b	P*_*TEF1*_*-***tHMG1*_*Sc*_**-T*_*ADH1*_*/P*_*PGK1*_*-***tHMG1*_*Xd*_**-T*_*CYC*_*	XI-1

**(B) β-Ionone production**			

**Strain**	**Parental Strain**	**Genotype**	**Integration Site (Chr-Site)**

β-iono2.1	β-Car2.1	P*_*PGK1*_*-***CCD1*_*Ph*_**-T*_*CYC*_*	XI-2
β-iono3.1	β-Car3	P*_*PGK1*_*-***CCD1*_*Ph*_**-T*_*CYC*_*	XI-2
β-iono4.1	β-Car4.b	P*_*PGK1*_*-***CCD1*_*Ph*_**-T*_*CYC*_*	XI-2
β-iono4.2	β-iono4.1	P*_*PGK1*_*-***CCD1*_*Ph*_**-T*_*CYC*_*	X-4
β-iono5.1	β-Car5	P*_*PGK1*_*-***CCD1*_*Ph*_**-T*_*CYC*_*	XI-2
β-iono5.2	β-iono5.1	P*_*PGK1*_*-***CCD1*_*Ph*_**-T*_*CYC*_*	X-4
β-iono5.3	β-iono5.2	P*_*PGK1*_*-***CCD1*_*Ph*_**-T*_*CYC*_*	XI-1

*The bold font represent the name of genes.*

**FIGURE 2:**
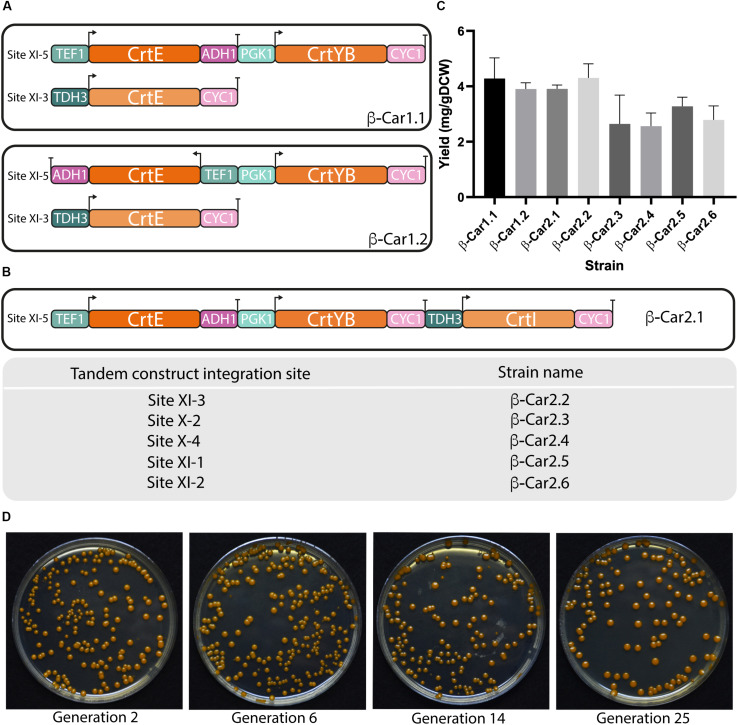
Design and evaluation of different transcriptional unit architectures and integration sites for carotenoids production in *S. cerevisiae*. General scheme of the different transcriptional architectures **(A)** and integration sites **(B)** evaluated for carotenoids production in yeast. **(C)** Total carotenoids yields achieved in shake flask cultivations after 72 h by strains with a single copy of the carotenogenic genes in different arrangements. The data represent the average and standard deviation of three independently grown cultures. **(D)** Color pigmentation of transformants remained stable throughout cultivations.

Optimal co-expression of two or more of these genes was evaluated by building two differentially oriented transcriptional units: Head-to-Head (termed bidirectional hereafter); and Head-to-Tail, (termed tandem hereafter). The CrtE and CrtYB genes were expressed following these two arrangements using the strong constitutive promoters TEF1 and PGK1, respectively, included in the bidirectional promoter plasmid library developed by Partow et al. (2010). The third CrtI gene was expressed under the TDH3 promoter and integrated in a different *locus*, completing the β-carotene biosynthetic pathway (Figure 2A). The two resulting strains β-Car1.1 (bidirectional) and β-Car1.2 (tandem) were cultured in shake flasks, reaching similar total carotenoids titers of 4 mg/gDCW and 3.8 mg/gDCW, respectively (Figure 2C). These results indicated that carotenoid production was unaffected by gene orientation, suggesting that their transcription did not interfere with each other. It is worthy to mention that we did not evaluate a convergent (Tail-to-Tail orientation) construct, since Carquet et al. (2015) reported that transcriptional units with two genes in this orientation dramatically reduced gene expression (mRNA) compared to the tandem arrangement, possibly as a consequence of transcription interference (TI) at the terminator sites.

Following the above results, we constructed a tandem cassette with the three carotenogenic genes. Adding a third gene to the transcriptional unit has the advantage of simultaneously expressing the heterologous pathway using only one integration site in the yeast genome. Comparison of the β-Car1.1 and β-Car2.1 - both in tandem configuration - revealed that the pXI-5 *locus* was mostly unaffected by the size of the DNA construct (from 5 kb with two genes to 7.7 kb with three genes), at least until this length (Figure 2C), since this new strain also reached 3.8 mg/gDCW of total carotenoids at 72 h.

The second variable analyzed was the location (i.e., the integration site) of the carotenogenic genes. Several, well-defined chromosomal integration sites, e.g., URA3, LEU2 and HO sites, have been widely employed in *S. cerevisiae* for the expression of heterologous genes (Flagfeldt et al., 2009; Westfall et al., 2012). Mikkelsen et al. (2012) identified eleven strong integration sites, strategically positioned between essential genes, which enable the construction of genetically stable strains with minimal risk of gene loss by recombination. However, the authors reported that these sites differed in their expression strength, adding another design variable for the construction of production strains. Consequently, we evaluated the expression capacity of six different *loci* (β-Car2.1 to β-Car2.6 strains), using cloning-compatible USER integrating plasmids with the three carotenogenic genes (CrtE/CrtYB/CrtI) displayed in tandem (Figure 2B). In order to avoid recombination between the different DNA parts, we employed three different promoters and two terminators - t_*CYC*_ and t_*ADH*__1_. Moreover, we noted that after several rounds of cell cultures, the color of the pigmented strains remained constant, pointing to the generation of genetically stable recombinant strains (Figure 2D).

The β-carotene yields of the βCar2.1 to βCar2.6 resulting strains, expressing the carotenogenic pathway in either one of the two *loci* used before (XI-3 or XI-5 integration sites), or in other four additional sites (X-2, XI-1, XI-2, and X-4) were compared (Figure 2C). According to the integration site of the tandem, total carotenoid yields ranged between 2.6 and 4.1 mg/gDCW after 72 h cultivation, a 60% difference between the integration sites with the highest (pXI-5) and the lowest (pXI-2) expression. Therefore, our results confirmed XI-5 and XI-3 *loci* as the strongest integration sites, in agreement with Mikkelsen et al. (2012). Although we only evaluated 6 of the 11 high expression sites reported by these authors, the former are sufficient for our construction purposes as they enable engineering up to 18 genes using three-gene tandem cassettes. Moreover, this number could be eventually increased, since it is possible to engineer up to 7 genes in just one marker less construct using CRISPR/Cas9 (Shi et al., 2016).

### Gene Dosage Tuning Significantly Impacts β-Carotene Accumulation

**FIGURE 3:**
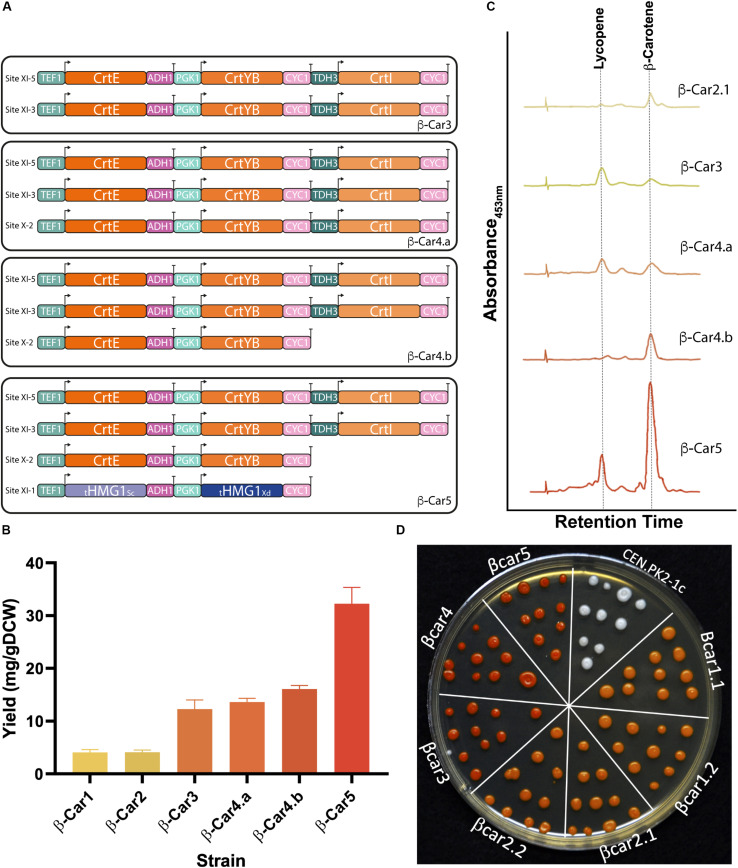
Tuning gene dosage for high accumulation of total carotenoids and β-carotene. **(A)** Evaluation of transcriptional units with different gene dosage. **(B)** Total carotenoid yields reached by strains with different dosage of carotenogenic genes after 72 h of cultivation in shake flasks. This strategy produced an 8-fold increase from the initial yield (from 4 mg/g DCW of β-Car1, to 32 mg/g DCW of β-Car5). The presented data represents the average and standard deviation of three independent cultures. **(C)** Carotenogenic profiles measured by HPLC after 72 h of cultivation (profiles are in the same scale). **(D)** Representative colonies of the constructed strains. Cells were plated after 24 h in YPD culture and the picture was taken 48 h later.

To further increase β-carotene yield, we integrated an extra copy of the three heterologous genes (tandem cassette) into the strain β-Car2.1, obtaining the β-Car3 strain (Figure 3A and Table 1). This strain produced up to 12 mg/gDCW of total carotenoids after 72 h cultivation in shake flasks, a 3-fold increase compared to the parental strain. No significant effect on cell growth (Figure 3B and Supplementary Table S3) was observed. While the increase in gene dosage improved the total carotenoids yield, an analysis of the carotenogenic profile revealed a higher lycopene content than the parental β-Car2 strain, which produced mainly β-carotene (Figure 3C). Indeed, in the β-Car3 strain, more than half of the total carotenoid content is lycopene, which still can be converted to β-carotene by the CrtYB enzyme.

Fine-tuning of the heterologous gene expression is likely the most difficult step during strain optimization, as the latter not only has to produce high amounts of the desired compound, but also avoid side reactions and toxicity. In our particular case, elevated lycopene concentrations impair normal yeast growth (López et al., 2015) and, therefore, the CrtI gene dosage – coding for the enzyme responsible of producing lycopene - has to be carefully tuned. Hence, we amplified only the first two genes from the tandem construct, CrtE and CrtYB, and then integrated them in the pX-2 site of the β-Car3 strain to promote phytoene production and lycopene consumption (Figure 3A). The resulting strain, β-Car4.b, produced a final yield of 16 mg/gDCW of total carotenoids after 72 h growth in shake flasks (Figure 3B). Moreover, the carotenoid profile showed an increased ratio of β-carotene to lycopene, although the strain still maintained a significant amount of residual lycopene. Notably, incorporation of the CrtI gene in this construct (see β-Car4.a in Figure 3A) was not beneficial for β-carotene synthesis; furthermore, it lowered final biomass concentration (Supplementary Table S3).

Finally, to further improve the carbon flux toward β-carotene, we integrated two extra copies of the truncated version of the HMG-CoA reductase gene (tHMGR1), one from *S. cerevisiae* and the other from *X. dendrorhous*, to avoid gene loss by recombination. The addition of several copies of this gene has been widely employed to increase the flux through the mevalonate (MVA) pathway in microbial cell factories, after this enzyme was identified as the major rate-limiting step of the pathway (Shimada et al., 1998; Wang and Keasling, 2002; Kampranis and Makris, 2012). The expression of these two genes in the β-Car4.b strain yielded the β-Car5 strain, which produced 32 mg/gDCW of total carotenoids (2-fold increase) (Figure 3B), with a higher ratio of β-carotene to lycopene (Figure 3C).

### The Extent of β-Carotene Accumulation Determines β-Ionone Final Titer

**FIGURE 4:**
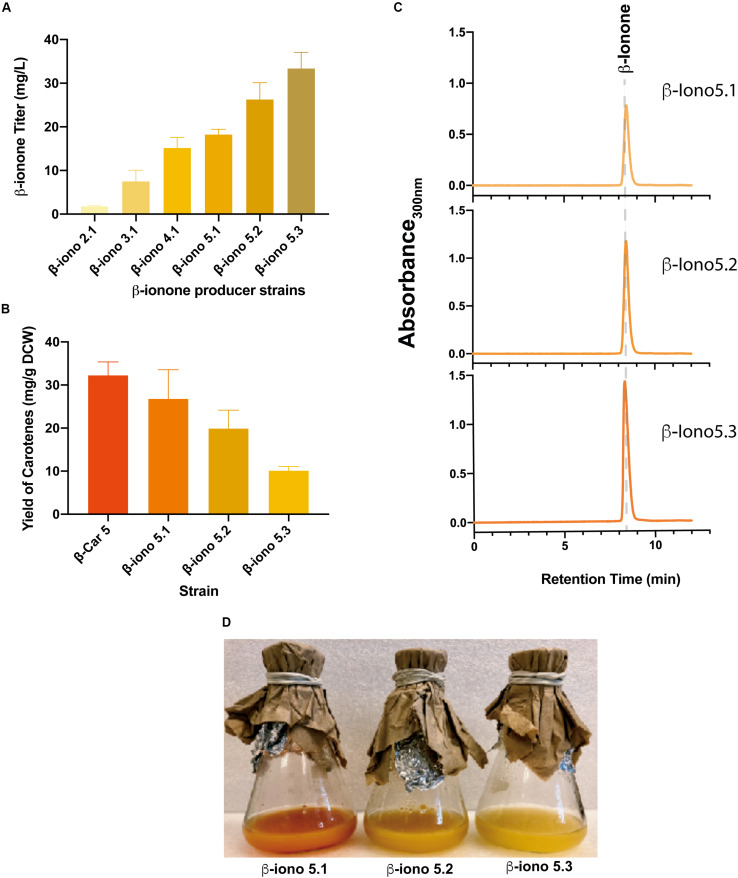
Integration of engineered CCD1 and evaluation of the gene dosage for the production of β-ionone. **(A)** β-ionone titer correlates with higher β-carotene accumulation of the parental recombinant strains. Similarly, the increase in the gene dosage of fyn-PhCCD1 improves β-ionone titers in the same manner. **(B)** The higher the β-ionone accumulation, the lower the amount of residual carotenoids. **(C)** Chromatograms of the β-iono5 strains show increasing β-ionone titters as the fyn-*Ph*CCD1 gene copy number increases. **(D)** Shake flasks cultures of strains with increasing fyn-*Ph*CCD1 gene dosage evidence overall higher β-ionone production as the dark orange color associated to carotenoid accumulation fades.

To evaluate β-ionone production under different β-carotene production conditions, we integrated a previously engineered CCD1 gene from *Petunia hybrida* (called fyn-*Ph*CCD1 hereafter) (Werner et al., 2019) into the different carotenogenic strains. Since the initial carotene producing strains (β-Car1.1-2 and β-Car2.1-6) displayed similar carotenoid yields, only the β-Car2.1 strain was transformed with the fyn-*Ph*CCD1 gene. Four new strains were constructed in a similar fashion, namely: β-iono2.1, β-iono3.1, β-iono4.1 and β-iono5.1, derived, respectively, from strains β-Car2.1, β-Car3, β-Car4.b, and β-Car5 (Figure 4A, refer to Table 1 for details).

Overall, the more β-carotene the strain accumulates, the higher the β-ionone titer (Figures 3B, 4C). For instance, the strain with the lowest total carotenoid accumulation (4 mg/gDCW in β-Car2.1) produced only 1.8 mg/L β-ionone after integration of fyn-*Ph*CCD1, whereas the strain with the highest carotenoid yield (32 mg/gDCW in β-Car5) reached 18.2 mg/L. The latter represents a 10-fold increase in the final β-ionone titer under the same culture conditions. The potential for further β-ionone production was also evaluated by measuring the residual content of carotenoids at the end of the cultivation (Figure 4B). The highest β-ionone (and β-carotene) producers – β-iono4.1 and β-iono5.1 – also exhibited the highest residual carotenoid content, 14.2 mg/gDCW and 26.7 mg/gDCW, respectively. Thus, there remains ample room for increasing β-ionone biosynthesis in β-iono5.1 strain, considering that approx. 18% of the total β-carotene was actually digested by the fyn-*Ph*CCD1 enzyme, according to our preliminary estimations. Finally, it is noteworthy that remnant carotenoids in these strains was mostly β-carotene, with no detectable traces of lycopene (Supplementary Figure S1).

### Incrementing the Gene Dosage of fyn-*Ph*CCD1 Boosts β-Ionone Production in β-Carotene Hyperproducing Strains

To further increase β-ionone production, we integrated an additional copy of the fyn-*Ph*CCD1 gene in the β-iono5.1 strain, resulting the β-iono5.2 strain. The same strategy was performed in the β-iono4.1 (resulting in the β-iono4.2 strain), displaying similar results as in the case of the β-iono5.2 strain (Supplementary Figure S2). The β-iono5.2 strain reached a higher β-ionone titer of 26.3 mg/L compared to β-iono5.1 (18.2 mg/L, Figure 4A). Given that there are still significant amounts of accumulated total carotenoids, a third copy of the fyn-*Ph*CCD1 gene was integrated into the β-iono5.2 strain. The resulting β-iono5.3 strain achieved more than 33 mg/L of β-ionone, a 19-fold improvement over the initial strain (β-iono2), and the highest β-ionone-producing *S. cerevisiae* strain reported to date (Figure 4A). The increased production of β-ionone by the β-iono5 strains resulting from increasing the fyn-*Ph*CCD1 gene copy number can be clearly evidenced in the chromatograms for each of the latter strains (Figure 4C) and the aspect of the culture broth (Figure 4D).

Our final *S. cerevisiae* production platform achieved a similar titer than previous reports for *E. coli* in shake flask cultures (up to 32.4 mg/L of β-ionone, Zhang et al., 2018). In the latter study, the authors used high copy number plasmids – arranged in modular fashion - to increase the carbon flux through a heterologous MVA pathway, thereby increasing the flux toward β-ionone. Notably, the employed plasmids can reach up to 10 copies per cell, which exceeds the number of gene copies presented here. In order to lessen the metabolic burden imposed by the high copy plasmids and achieve high β-ionone concentrations, an inducible expression system was implemented that enabled decoupling production from growth (Zhang et al., 2018). However, such plasmids can suffer from genomic instability issues (Ryan et al., 2014) or lack of reproducibility in industrial fermentations (Rugbjerg and Sommer, 2019). In contrast, our strategy – though more conservative – is particularly robust and suited for cases where production is balanced and metabolic toxicity is not an issue. Importantly, if necessary, our platform can be readily adapted for inducible expression to decouple production from growth, and/or to down-regulate competing pathways.

Finally, we noted that both, the increase in β-ionone production and the decrease in total carotenoids accumulation, followed a nearly linear trend, which highlights that fyn-*Ph*CCD1 is likely capacity-limited, i.e., maximum catalytic rate (V_*max*_) has been reached. However, the amount of β-carotene cleaved experimentally by fyn-*Ph*CCD1 does not correspond to the optimal stoichiometric amounts of produced β-ionone, i.e., 1 mol of β-carotene produces 2 mol of β-ionone. In order to gain further insights about the fate of β-carotene in β-iono strains, a genome-scale metabolic model was constructed and interrogated.

### Genome-Scale Stoichiometric Analysis Suggests fyn-*Ph*CCD1 Has Highly Unspecific Cleavage Activity During β-Ionone Production

In order to better understand β-ionone production in yeast, we tailored an existing Genome-Scale Metabolic Model (GSMM) of *S. cerevisiae* for predicting the (apo)carotenoids yields for each producing strain. To this task, the curated *i*MM904 GSMM (Mo et al., 2009) was constrained for accurately representing aerobic growth with glucose as carbon source, as well as the production of the measured carotenoids. The details of the contextualization method can be found in the Methods section.

**FIGURE 5:**
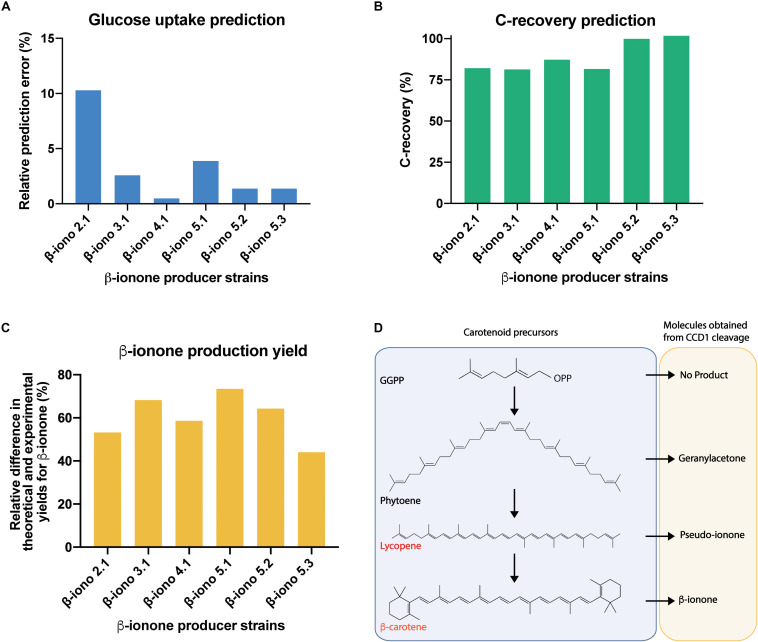
Genome-scale stoichiometric analysis shows high degree of carbon balance closure in β-iono mutant strains and points to unspecific cleavage activity of *Ph*CCD1 for the reduced β-ionone yields observed. Evaluation of the prediction fidelity of the GSMM for optimal specific glucose uptake under culture conditions **(A)** and carbon recovery for all producer strains **(B)**. **(C)** Relative difference in the maximum predicted and experimental yield of β-ionone under conditions of high carbon balance closure. **(D)** Proposed apocarotenoids generated by the cleavage of the CCD1 enzyme on different carotenoids.

Before examining the carotenoid production profile, we evaluated the prediction power of the contextualized GSMM (Figures 5A,B). To this task, the GSMM was constrained to reproduce the observed biomass, β-ionone, and β-carotene yields for the different production strains (Figures 4A,B), and the specific growth and carbon dioxide rates reported for *S. cerevisiae* strains growing aerobically on glucose (Van Hoek et al., 1998). Using this data, the optimal (minimum) specific glucose uptake rate was computed for all the strains, and its value compared with the available experimental data (Van Hoek et al., 1998). This exercise amounts to optimize the biomass yield on glucose. Results showed excellent agreement between predictions and experimental measurements with a maximum relative error of approx. 10% (β-iono2.1 strain, Figure 5A), and an average deviation of approx. 3.3% (Figure 5A). Given these promising results, we next computed the degree of carbon recovery predicted by the GSMM using this reduced set of measurements. We note that calculation of this quantity is critical to properly interpret subsequent results about the (apo)carotenogenic production flux profile, as a low degree of closure (<80%) precludes drawing meaningful conclusions. Notably, in spite of the limited amount of information, the contextualized GSMM was able to account for most of the consumed carbon by β-iono2.1, β-iono3.1, β-iono4.1, and β-iono5.1 (> 80% and up to 87% in β-iono4.1, Figure 5B), and in the case of the highest producers - β-iono5.2 and β-iono5.3 -, it was almost able to close the carbon balance (approx. 100% closure, Figure 5B).

The previous results indicated that the GSMM has a high prediction fidelity and, more importantly, it can be safely employed as a prospecting tool for gaining insights about the carbon redistribution in the β-ionone production pathway. In particular, it is of interest to determine how much of the β-carotene is cleaved and consumed by fyn-*Ph*CCD1 for producing β-ionone, relative to other possible (apo)carotenoid products. Indeed, this class of enzymes can act on multiple carotenoid substrates (e.g., ζ-carotene, γ-carotene, lycopene, β-carotene, among others) and produce several apocarotenogenesis products (e.g., geranylacetone, pseudoionone, β-ionone, among others) (Vogel et al., 2008) (see Figure 5D). Therefore, we used the contextualized GSMM to estimate the maximum production yield of β-ionone under the assumption that β-carotene is only being diverted to β-ionone and accumulated in the amounts observed experimentally. To this task, we calculated maximum β-carotene production under the experimental conditions, but without the requirement of β-ionone production. The difference between the theoretical and experimental β-carotene accumulation then represents the carbon that could have been allocated for achieving the maximum β-ionone yield. Lastly, we employed this quantity to constrain the carbon flux toward β-carotene formation and maximized the β-ionone yield. If all the carbon that could be allocated toward β-ionone coincides with the experimental production values, then the fyn-*Ph*CCD1 enzyme is producing β-ionone in the optimal stoichiometric amounts. This is, however, not the case for any of the β-iono strains (Figure 5C). Departures from the optimal β-carotene cleavage by fyn-*Ph*CCD1 range from 44% up to 73.4%, which suggests a significant limitation in this enzyme. In fact, in the optimal scenario, fyn-*Ph*CCD1 produces the C14 compound derived from the double cleavage of β-carotene in a molar ratio ranging from 0.15 (β-iono5.1) to 0.32 (β-iono5.3) per mol of β-ionone, which is far from the optimal stoichiometric ratio of 1:2 (refer to Supplementary File Presentation 1). As the carbon mass balance must close, this necessarily implies that the fyn-*Ph*CCD1 is either cleaving the β-carotene at only one site and/or acting on other upstream substrates. Further experimental efforts should be then focused on measuring fyn-*Ph*CCD1 side reactions products to experimentally validate this hypothesis.

The engineered CCD1 employed in this work has indeed increased accessibility of other known lipophilic substrates for this enzyme (Werner et al., 2019), which offers support for the model suggestions. To tackle this limitation, site-directed mutagenesis may be applied to improve the selectivity and activity of *Ph*CCD1, thereby avoiding undesirable side-products, such as pseudo-ionone or geranyl acetate, and favoring ionone production. However, given the high similarity between the possible reactants and the highly conserved active site of this enzyme (Werner et al., 2019), it is likely that this strategy will be plagued with obstacles. Another plausible alternative is the fusion of key enzymes. Spatial proximity and the adequate disposition of the enzymes inside of a metabolic pathway is critical for the efficient conversion of intermediates into the final product. For instance, the CCD1 could be fused to the lycopene cyclase domain of the CrtYB, avoiding the access to other intermediates. A recent report of heterologous production of β-carotene in *S. cerevisiae* has shown promise for the optimization of carotenoids in yeast (Rabeharindranto et al., 2019).

## Conclusion

In this work, we developed several recombinant *S. cerevisiae* strains, capable of producing increasing amounts of the apocarotenoid β-ionone and its precursor, β-carotene. To this task, we employed a systematic approach where different variables involved in the constitutive expression of recombinant genes were optimized, namely: the architecture of the heterologous transcription units, the location of the integration site, and the dosage of key genes needed for β-ionone synthesis. Our results demonstrated that high β-ionone production can be attained by implementing rational metabolic engineering strategies. Systematic assessment of the above variables led to the construction of recombinant strains capable of producing 32 mg/gDCW of total carotenoids (β-Car5), and 33 mg/L of β-ionone (β-iono5.3). While this study presents the highest β-ionone titers reported so far in *S. cerevisiae* using shake flasks cultures, there is still room for improvement. Achieving a balanced gene expression for optimal production of the heterologous pathway is critical for reaching even higher yields. Subsequent studies should be focused at fine-tuning the expression of relevant genes by directly measuring transcriptional/protein data and metabolic production performance. Another possible strategy for improving this system is the implementation of an inducible system, where the expressions of (apo)carotenogenic and competitive pathways (e.g., sterol pathway) are decoupled is a promising approach. Finally, our experimental and modeling results confirmed that β-ionone production in yeasts is limited by the CCD1 efficiency in high-accumulating β-carotene strains. Further protein engineering efforts are thus needed to increase the overall efficiency of heterologous β-ionone conversion. Fusion enzymes and enzyme redirection could be attractive strategies to tackle this obstacle.

## Materials and Methods

### Genes and Plasmids

The genes used in this study were amplified by PCR from genomic DNA of *X. dendrorhous* (CrtYB, CrtE, CrtI and tHMGR1) and *S. cerevisiae* (tHMGR1). The gene coding for *fyn*-CCD1 of *P. hybrida* was kindly facilitated by Dr. Werner, where the peptide fyn and a linker were fused to the N-terminal of the full length *Ph*CDD1 gene (Werner et al., 2019). The TDH3 promoter was amplified by PCR from the episomal plasmid p426GDP.

Yeast integrating expression vectors were constructed using the Gibson Assembly method (Gibson et al., 2009) using the plasmid library developed by Mikkelsen et al. (2012). Genes and backbone vectors were PCR-amplified by Phusion High-Fidelity DNA polymerase (Thermo Scientific, Waltham, MA, United States), following the manufacturer’s instructions. The resulting PCR products were purified using the Wizard SV Gel and PCR Clean-Up System kit (Madison, Wisconsin, United States). Purified DNA fragments were then mixed with 1.33x Gibson master mix (isothermal buffer, T5 exonuclease 1 U/μL, Phusion DNA polymerase 2 U/μL, and Taq DNA ligase 40 U/μL) in 10 μL of final volume and incubated for 60 min at 50°C. The reaction products were transformed in *E. coli* Top10 cells (Thermo Fisher Scientific, United States). The assembled plasmids were purified using E.Z.N.A plasmid mini Kit (Omega Bio-tek, United States) and verified by sequencing (Macrogen Inc., South Korea). Primers used for all amplifications are in Supplementary Table S1.

Depending on the transcriptional unit architecture, different construction workflows were employed. The bidirectional, head to head (HH), plasmid XI-5 HH YB/E was constructed as follows; pXI-5 plasmid was amplified to assemble in first place the CrtYB gene under the PGK1 promoter and CYC1 terminator. This plasmid was named XI-5 CrtYB. Then, XI-5 CrtYB was amplified to assemble the CrtE gene under TEF1 promoter and ADH1 terminator. The tandem architecture, head to tail (HT), of this last plasmid was constructed by the amplification of the transcriptional unit containing the CrtE gene, arranged in the same direction than the CrtYB gene. This plasmid was designated XI-5 HT YB/E. On the other hand, the XI-3 CrtI plasmid was built in two steps. Firstly, the TDH3 promoter was assembled into the pXI-3 plasmid, which was designated XI-3 TDH3. Then, the CrtI coding gene was inserted into the XI-3 TDH3 plasmid.

Finally, for the construction of XI-5 HT tHSc/tHXd and HT YB/E/I plasmids, the XI-5 HT YB/E was used as backbone. For XI-5 HT tHSc/tHXd, the XI-5 HT YB/E plasmid was used to assemble the coding gene tHMG1R of *X. dendrorhous* (under PGK1 promoter). This plasmid was named XI-5 HT tHXd/E. The latter was then amplified for inserting the coding gene tHMG1R from *S. cerevisiae* (under TEF1 promoter). For the construction of the HT YB/E/I plasmid, the XI-5 HT YB/E plasmid was opened by PCR and the transcriptional unit XI-3 CrtI amplified and assembled into the open plasmid XI-5 HT YB/E. The XI-3 CCD1 plasmid was constructed by replacing the CrtI gene of the XI-3 CrtI plasmid by the PhCCD1 gene. For the construction of the HT tHSc/tHXd/CCD1 plasmid, transcription units of the CrtYB and CrtE genes HT YB/E/I was replaced by the tHSc/tHXd construct from the XI-5 HT tHSc/tHXd plasmid. This yielded the HT tHSc/tHXd/I plasmid. In this plasmid, the CrtI gene was replaced with the *Ph*CCD1 gene from XI-3 CCD1 plasmid.

### Yeast Strains Construction

The CEN.PK2-1c parental strain (MATa, *ura3-52 trp1-289 leu2-3, 112his3*Δ) was used in this study. The constructed strains are listed in Table 1. These strains were built using CRISPR/Cas9 method, following the protocol from Tom Ellis lab, which is freely available on the Benchling webpage^1^. The plasmids used in this study are available on Addgene, whereas the gRNAs were obtained from the USER library (Supplementary Table S2).

The β-Car strains were transformed following the same protocol, but using different integration constructs and primers, depending on the transcriptional architecture. In the following, the β-Car1 protocol is described for illustration. The protocol details for remaining β-Car strains can be found in Supplementary Table S1. The β-Car1 strain was built by the amplification of the XI-5 HH YB/E vector with the primers p1/p2 to be integrated on the pXI-5 locus of the CEN.PK strain. All the DNA fragments obtained by PCR had 40 bp homology to ensure integration into the genome. PCR protocols and product purification were executed as described before for plasmid construction. The transformants were selected using SC-URA plates (1.8 g/L yeast nitrogen base, 5 g/L ammonium sulfate, 0.8 g/L CSM-Ura mixture, 20 g/L of glucose, and 20 g/L of Bacto-agar). Only colored colonies were isolated and grown on YPD plates (1% yeast extract, 2% peptone, 2% glucose, and 1% Bacto-agar). One of the transformants was transformed with the XI-3 CrtI vector, which was amplified using primers p3/p4, to be integrated into the pXI-3 locus. The transformants were selected on SC-LEU (1.8 g/L yeast nitrogen base, 5 g/L ammonium sulfate, 0.8 g/L CSM-Leu mixture, 20 g/L of glucose, and 20 g/L of Bacto-agar). The correct integration into the pXI-3 locus was verified using primers for XI3F/XI3R. The positives colonies were grown on YPD plates.

Initial β-iono strains (2, 3, 4b, and 5) were generated by transformation of the corresponding β-Car strains. To this task, the XI-3 CCD1 plasmid was amplified using primers p9/p10 and integrated into the pXI-2 locus. The transformants were selected on SC-URA plates and positive colonies were grown on YPD plates. Correct cassette integration was verified by PCR using primers XI2F/XI2R. β-iono4.1 and β-iono5.1 strains were constructed by transformation of the β-Car4 and β-Car5 strains with the XI-3 CCD1 plasmid amplified using primers p11/p12 and integrated into the pX-4 locus. Transformants were selected on SC-LEU plates and positives colonies were grown on YPD plates. As before, PCR was performed to verify correct integration into the locus using primers X4F/X4R. Finally, the β-Iono5.1 strain was transformed with HT tHSc/tHxd/CCD1, which replaced the construct HT tHSc/tHXd on locus pXI-1. This yielded the β-iono5.2 strain, which was selected for positive transformants on SC-HIS (1.8 g/L yeast nitrogen base, 5 g/L ammonium sulfate, 0.8 g/L CSM-His mixture, 20 g/L of glucose, and 20 g/L of Bacto-agar). Again, correct integration was verified by PCR using primers CCD1F/XI1R.

### Growth Conditions

Single colonies were inoculated in 3 mL pre-cultures in YPD medium (1% yeast extract, 2% peptone and 2% glucose). β-carotene-producing strains were grown in 250-mL shake flask cultures at 30°C and 170 rpm in a horizontal shaker with 20 mL of YPD medium. In the case of β-ionone producing strains, cultures were grown with 18 mL of YPD medium and a dodecane phase (10%v/v). All shake flask cultures were inoculated from pre-cultures to an initial OD_600_ of 0.1 (0.04 gDCW/L).

### β-Ionone Quantification

Cultures were centrifuged for 10 min at 6000 rpm on 50-mL tubes. The organic phase was collected in 500-μL glass vials for subsequent analysis. β-ionone quantification was performed by HPLC LaChrom (Merk-Hitachi) coupled to a diode array detector, using a C30 YMC Carotenoid column (5 μm, 150 × 10 mm) (YMC, Japan). A mobile phase of 2-propanol with a 1 mL/min flow under isocratic condition was employed for elution. β-ionone was detected at 330 nm and 8.39 min retention time. Concentrations of β-ionone were estimated using a calibration curve with external standards with 12 to 378 mg/L linear range (refer to Supplementary Figure S3 for more details).

### Total Carotenoids Extraction and Profile Analysis

Carotenoid extraction was carried out after 72 h of cultivation. 250 μL of culture were centrifuged for 1 min at 13,000 rpm, the supernatant was discarded, and cell pellets broken with 500 μL of 0.5-mm glass beads with 1 mL dodecane in a homogenizer (Benchmark Scientific, NJ, United States) using 5 cycles of 90 s at 3,500 rpm. The cell-beads mixture was then centrifuged at 13,000 rpm for 2 min, and total carotenoids in the dodecane phase were quantified by spectrophotometry (Thermo Fisher, Waltham, MA, United States) at 453 nm. Total carotenoid concentration was estimated using a calibration curve of pure β-carotene (Carotenature, Switzerland), with 1 to 6 mg/L linear range. Samples above this range were diluted appropriately.

For determination of the carotene profile, the previous protocol was executed, but instead of dodecane, cells were broken using 1 mL of acetone. Carotenoids were then separated by RP-HPLC using a reverse phase RP18 column (5 μm, 4.6 × 125 mm) (Merck, Darmstadt, Germany). A mixture of acetonitrile:methanol:isopropyl (85:10:5 v/v) was employed as mobile phase with a 0.5 mL/min flow under isocratic conditions. The elusion spectrum was recovered using a diode array detector.

### Genome-Scale Stoichiometric Analysis

To probe β-ionone production in yeast, an existing Genome-Scale Metabolic Model (GSMM) of *S. cerevisiae* was contextualized for predicting the different (apo)carotenoids production of the engineered strains. To this task, the curated *i*MM904 GSMM (Mo et al., 2009) was constrained following recent guidelines for accurately describing aerobic growth with glucose as sole carbon source in yeasts (Pereira et al., 2016; Torres et al., 2019). Stoichiometric calculations were performed using constrained-based methods from COBRA Toolbox v3.0 (Heirendt et al., 2019) within the MATLAB 2017a environment (The MathWorks, Natick, MA). The model and employed scripts are available in the Supplementary File Presentation 1.

## Data Availability Statement

All datasets presented in this study are included in the article/Supplementary Material.

## Author Contributions

JL and DB constructed the strains and performed shake flask cultures. CC developed the methods for the quantification of carotenoids and apocarotenoids and analyzed the results. JL, DB, and NA constructed the plasmid library used in this study. PAS developed the genome-scale metabolic model used for the apocarotenoid analysis. JL, DB, and PAS participated in the design, coordination of the study, and draft of the manuscript. EA supervised the whole research and revised the manuscript. All authors read and approved the final manuscript.

## Conflict of Interest

JL was employed by the company DICTUC S.A. EA is an advisor for DICTUC S.A. The remaining authors declare that the research was conducted in the absence of any commercial or financial relationships that could be construed as a potential conflict of interest.

## Footnotes

1 https://benchling.com/pub/ellis-crispr-tools
