# Supplementary material for: Engineering Saccharomyces cerevisiae for the Overproduction of β-Ionone and Its Precursor β-Carotene
Source: Front Bioeng Biotechnol. 2020 Sep 30;8:578793. doi: 10.3389/fbioe.2020.578793 (PMC7556307; doi:10.3389/fbioe.2020.578793)
Supplement: Supplementary file 1 [file Data_Sheet_1.docx]

**Supplementary Material**

**Engineering *Saccharomyces cerevisiae* for the overproduction of β-ionone and its precursor β-carotene**

**Javiera López^1#^, Diego Bustos^2#^, Conrado Camilo^1^, Natalia Arenas^2^, Pedro A. Saa^2^, Eduardo Agosin^1,2*^**

1. Centro de Aromas & Sabores, DICTUC S.A., Macul, Santiago, Chile.
2. Department of Chemical and Bioprocess Engineering, School of Engineering, Pontificia Universidad Católica de Chile, Santiago, Chile.

^*^Corresponding author: [agosin@ing.puc.cl](mailto:agosin@ing.puc.cl)

^#^Both authors contributed equally to this work

**Supplementary Table 1: List of the primers used in the DNA constructs**

| **NAME** | **SEQUENCE 5’-3’** |
| --- | --- |
| BB_CrtYB_Fw | ACAAAGCGATACGTAAAAAAATGACGGCTCTCGCATATTAC |
| CrtYB_BB_Rv | AGCGGATGAATGCACGCGATTTACTGCCCTTCCCATCC |
| CrtYB_BB_Fw | GCGGATGGGAAGGGCAGTAAATCGCGTGCATTCATCCG |
| BB_CrtYB_Rv | TAATATGCGAGAGCCGTCATTTTTTTACGTATCGCTTTGTTTTATATTTG |
| CrtE_BB_Fw | CCATTTTTTTACCCGTTGATTTGTAATTAAAACTTAGATTAGATTGCTATGCT |
| BB_CrtE_Rv | TAGCCGATATCCCTCGTGAATCGCACGCATTCCGTTG |
| BB_CrtE_Fw | ACCAACGGAATGCGTGCGATTCACAGAGGGATATCGGC |
| CrtE_BB_Rv | ACAAATCAACGGGTAAAAAAATGGATTACGCGAACATCC |
| ADH1_BB_Fw | CAGGTATAGCATGAGGTCGCTCCGGCCGGCCTGGAAGTAC |
| BB_TEF1_Rv | CATTTTGAAGCTATGGTGTGTGATCGCACGCATTCCATGCG |
| BB_TEF1_Fw | CTCGCATGGAATGCGTGCGATCACACACCATAGCTTCAAAATGTTTC |
| ADH1_BB_Rv | AGGTACTTCCAGGCCGGCCGGAGCGACCTCATGCTATACC |
| BB_TDH3_Fw | ACTCTAGAGGATCCCCGGGTACCCAGTTCGAGTTTATCATTATCAATACTCGCCATTTC |
| TDH3_BB_Rv | TGGATCCACTAGTTCTAGAATCCGTCGAAACTAAGTTCTGG |
| BB_TDH3_Rv | ATCCTCTAGAGTCGACCTGCAGGCGGCCACCGCACGCATTCCGTTGGTAG |
| TDH3_BB_Fw | ATTCTAGAACTAGTGGATCCATCGCGTGCATTCATCCG |
| BB_CrtI_Fw | ATTCTAGAACTAGTGGATCCATGGGAAAAGAACAAGATCAGG |
| CrtI_BB_Rv | AGCGGATGAATGCACGCGATTCAGAAAGCAAGAACACCAAC |
| CrtI_BB_Fw | TTGGTGTTCTTGCTTTCTGAATCGCGTGCATTCATCC |
| BB_CrtI_Rv | CTGATCTTGTTCTTTTCCCATGGATCCACTAGTTCTAGAATCC |
| TDH3_CrtI_BB_Fw | CTGAAAACCTTGCTTGAGAAATCGCGTGCATTCCCGGC |
| BB_TDH3_CrtI_Rv | GTCGACCTGCAGGCGGCCACCTTCGAGCGTCCCAAAAC |
| BB_TDH3_CrtI_Fw | AGGTTTTGGGACGCTCGAAGGTGGCCGCCTGCAGGTCG |
| TDH3_CrtI_BB_Rv | AGGCCGGGAATGCACGCGATTTCTCAAGCAAGGTTTTCAGTATAATG |
| BB_tHMG1Xd_Fw | ACAAAGCGATACGTAAAAAAATGGGCCACAAGAACCAG |
| tHMG1Xd_BB_Rv | AGCGGATGAATGCACGCGATTCAGGCATCCACGTTCAC |
| tHMG1Xd_BB_Fw | GTGTGAACGTGGATGCCTGAATCGCGTGCATTCATCCG |
| BB_tHMG1Xd_Rv | TTCTGGTTCTTGTGGCCCATTTTTTTACGTATCGCTTTGTTTTATATTTG |
| BB_tHMG1Sc_Fw | CAACGGGTAAAAAAAAAACAATGGCTGCAGACCAATTG |
| tHMG1Sc_BB_Rv | CCAACGGAATGCGTGCGATTTTAGGATTTAATGCAGGTGACGG |
| tHMG1Sc_BB_Fw | CACCTGCATTAAATCCTAAAATCGCACGCATTCCGTTG |
| BB_tHMG1Sc_Rv | ACCAATTGGTCTGCAGCCATTGTTTTTTTTTTACCCGTTGATTTG |
| BB_CCD1_Fw | TTAGTTTCGACGGAAGCGATACGTAAAAAAATGGGTTGTGTTCAGTG |
| CCD1_BB_Rv | TTAGAGCGGATGAATGCACGCGATTCATAACTTGGCTTGTTCTTGTATTT |
| CCD1_BB_Fw | AAGAACAAGCCAAGTTATGAATCGCGTGCATTCATCCG |
| BB_CCD1_Rv | TTACACTGAACACAACCCATTTTTTTACGTATCGCTTCCGTC |
| CYC_BB_Fw | AGGTTTTGGGACGCTCGAAGGTGGCCGCCTGCAGGTCGACTCTAGAGG |
| CYC_BB_Rv | CTAGAGTCGACCTGCAGGCGGCCACCTTCGAGCGTCCCAAAACC |
| p1 | CCACACTAGCCTTCGATTTGACACATCTCTAAGCTGAAACATTAAGTCCTCAGCGAGCTC |
| p2 | GTTGTGGGCAATTGGGTGTACTATGAAGCAGCCAATACCTGATCCCCGGGAATTGCCATG |
| p3 | TTTCCGTTTTAGGATATTGACGCCAAGCGTGCGTCTGATTATTAAGTCCTCAGCGAGCTC |
| p4 | AGTTTAATAATGATCTGTATTGCTGGCTCAATCCACGTAAGATCCCCGGGAATTGCCATG |
| p5 | ATTTCTTTTTCCTCGGGCAGAGAAACTCGCAGGCAACTTGATTAAGTCCTCAGCGAGCTC |
| p6 | ATGGGTAACGGGATCCCTCTGTGAGGGCCGATTATGCAGGGATCCCCGGGAATTGCCATG |
| p7 | TTCTTCCACGGAATACCAAGCCCATTGCAATGCGATGTTAATTAAGTCCTCAGCGAGCTC |
| p8 | GCAGCATTTAGCAGAGATTTGCCAATGCCAAGAAACTCCAGATCCCCGGGAATTGCCATG |
| p9 | CCATGAACTTCCACAAGTAAAGCTCGTTGACCAGTTGATCATTAAGTCCTCAGCGAGCTC |
| p10 | CTTTTTAATATCTGAAAGCGCTAGTCGTGTGTACCCCCCTGATCCCCGGGAATTGCCATG |
| p11 | GAGGGAACACTGGGGCAATAGGCTGTCGCCATTCAAGAGCATTAAGTCCTCAGCGAGCTC |
| p12 | TATTGTAATTCAAAAAAAAAAAGCGAATCTTCCCATGCCTGATCCCCGGGAATTGCCATG |
| XI3F | GCGTTTATTGTCGCATCGCTAGC |
| XI3R | GCAAGTTCTGTGGAGTTCCATGT |
| X2F | CTCGGAGATGGCGCATCTATTTG |
| X2R | AGTCGCCCGGTTGTTGAAA |
| XI1F | TGTATACGGTTTTAGATGCAGTATG |
| XI1R | ACAGCTTGGTCCATAGTTCTAG |
| XI2F | CGCAAAACTCAGTAATAAGCTTTCTG |
| XI2R | CGCACCAAAAGTAAGAAACG |
| X4F | AGAGAATTAGTATGGTACAGGATACG |
| X4R | ACACGTATTCTTGTGCACACG |
| CCD1F | ATGGGTAGAAAAGAATCAGATGAC |
| gRNAXI.1 | TGCTAAATGCTGCGTACAGA |

**Supplementary Table 2: Description of the plasmids used in this study**

| **Name** | **Plasmid description** |
| --- | --- |
| pWS082 | sgRNA entry vector |
| pWS158 | Cas9-sgRNA 2 micron vector with URA3 yeast marker |
| pWS171 | Cas9-sgRNA 2 micron vector with LEU2 yeast marker |
| pWS172 | Cas9-sgRNA 2 micron vector with HIS3 yeast marker |
| pWS173 | Cas9-sgRNA 2 micron vector with KanR yeast marker |
| pXI-5 | URA3-based integration plasmid carrying a bidirectional PTEF1-PPGK1 promoter and regions for homologous recombination |
| pXI-3 | URA3-based integration plasmid carrying a bidirectional TADH1-TCYC1 terminator and regions for homologous recombination |
| p426GPD | URA3-based expression plasmid carrying a pGPD promoter |
| XI-5 YB | PPGK1-CrtYB-TCYC1 |
| XI-5 HH YB/E | bidirectional PTEF1-CrtE-TADH1/PPGK1-CrtYB-CYC1 |
| XI-5 HT YB/E | Head to Tail promoter disposition of HH YB/E plasmid |
| XI-3 TDH3 | pXI-3 plasmid with TDH3 promoter |
| XI-3 CrtI | PTDH3-CrtI |
| HT YB/E/I | PTEF1-CrtE-PPGK1-CrtYB-PTDH3-CrtI Head to Tail promoter |
| HT tHSc/tHXd | PTEF1-tHMG1Sc-PPGK1-tHMG1Xd Head to Tail promoter |
| XI-3 CCD1 | PTDH3-PhCCD1 |
| HT tHSc/tHXd/CCD1 | PTEF1-tHMG1Sc-PPGK1-tHMG1Xd-PTDH3-CCD1 Head to Tail promoter |

**Supplementary Table 3: Final biomass concentration for each constructed strains after 72 h cultivation in 125 mL shake flasks.**

| **Strain** | **OD_600_** |
| --- | --- |
| CEN.PK2-1c | 20 |
| β-Car1.1/β-Car1.2 | 10.6 |
| β-Car2.1 | 9.0 |
| β-Car2.2 | 8.6 |
| β-Car2.3 | 12.3 |
| β-Car2.4 | 14.1 |
| β-Car2.5 | 12.7 |
| β-Car2.6 | 15.2 |
| β-Car3 | 11.1 |
| β-Car4.a | 10.2 |
| β-Car4.b | 12.1 |
| β-Car5 | 12.7 |
| β-Iono2.1 | 14.8 |
| β-Iono3.1 | 15.1 |
| β-Iono4.1 | 16.2 |
| β-Iono4.2 | 14.5 |
| β-Iono5.1 | 14.8 |
| β-Iono5.2 | 18.4 |
| β-Iono5.3 | 18.9 |

**
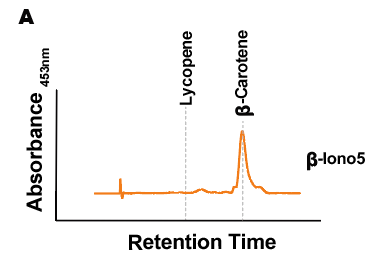
**

**Supplementary Figure 1.** HPLC profile of the distinct carotenoids present in the β-Iono5.1 strain.


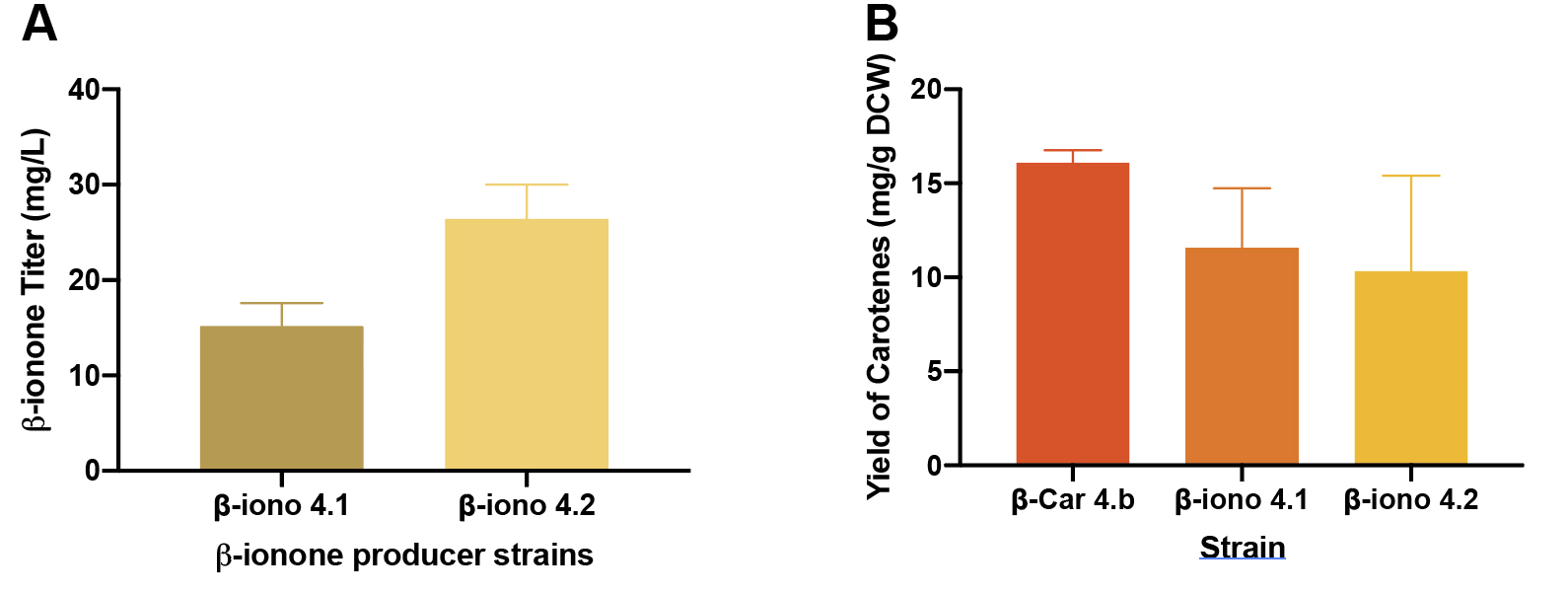


**Supplementary Figure 2.** (**A**) β-ionone titers reached adding a single copy and two copies of the *Ph*CCD1 gene to the β-Car4.a strain. (**B**) Residual carotene yielding after 72 hours cultivation in shake flasks of the same strains.


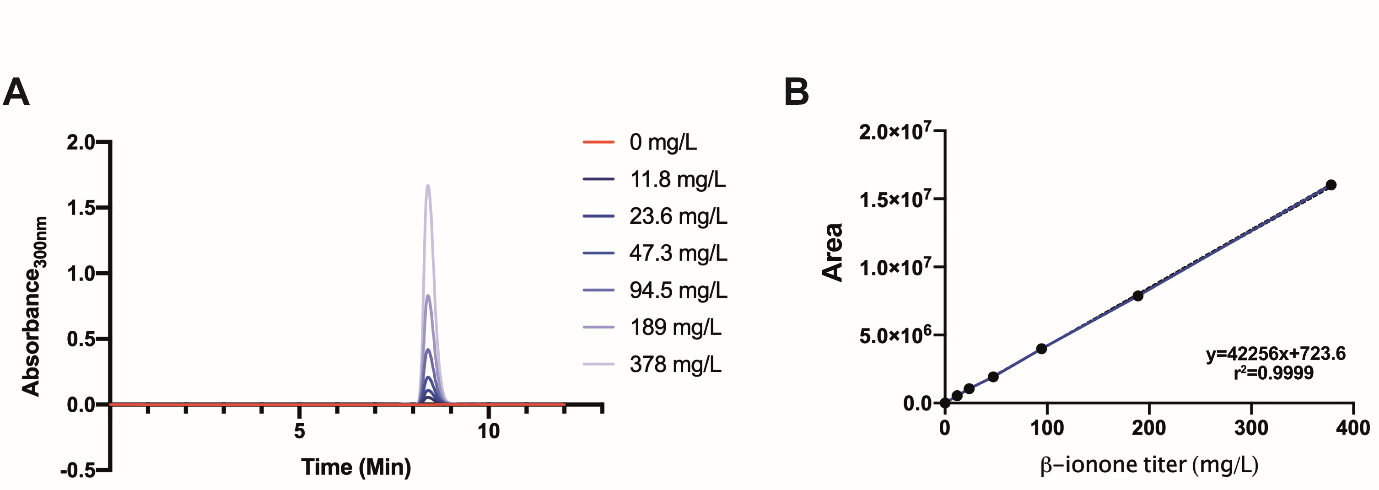


**Supplementary Figure 3.** Calibration curve for the quantification of β-ionone in HPLC at 300 nm. (**A**) Chromatograms for different β-ionone (standard) injections at various concentrations. The observed peaks coincided and displayed a retention time of 8.39 min in this method. (**B**) There is a perfectly linear correlation (R^2^ = 0.999) between the injected β-ionone concentration and the area under the peak curve.
